# Continuous Active Motion Versus Continuous Passive Motion for Rehabilitation of Patients After Total Knee Arthroplasty: A Systematic Review and Meta-Analysis

**DOI:** 10.1007/s43465-025-01681-2

**Published:** 2026-01-06

**Authors:** Claudia Sasse, Jonathan Lettner, Nikolai Ramadanov, Aleksandra Królikowska, Roland Becker, Robert Prill

**Affiliations:** 1 Center of Orthopaedics and Traumatology, Brandenburg Medical School Theodor Fontane, University Hospital Brandenburg, Brandenburg/Havel, Germany; 2 Physiotherapy Research Laboratory, Faculty of Physiotherapy, University Centre of Physiotherapy and Rehabilitation, Wroclaw Medical University, Wroclaw, Poland

**Keywords:** CAM, CPM, Physical therapy, Knee replacement, Range of motion, TKR

## Abstract

**Introduction:**

Rehabilitation following total knee arthroplasty (TKA) includes various therapeutic interventions, such as continuous active motion (CAM) and continuous passive motion (CPM). This meta-analysis aimed to compare the effects of CAM and CPM on pain, function, and range of motion post-TKA, focusing on the early postoperative period.

**Methods:**

A systematic literature search was conducted in databases including Medline, Cochrane Library, Embase, and CINAHL to identify randomized controlled trials (RCTs) comparing CAM and CPM post-TKA. Studies were required to include at least two postoperative measurements and combine the interventions with standard physiotherapy. The methodological quality of the included studies was assessed using the JBI appraisal tool, and statistical analysis was performed on suitable studies.

**Results:**

Seven RCTs involving 501 patients were included. Meta-analysis indicated significant pain reduction and improved functional outcomes with CAM compared to CPM, particularly in postoperative pain assessments. CAM showed advantages in functional tests, including the Sit-to-Stand and Timed-Up-and-Go tests. However, no significant differences were found for active and passive range of motion (ROM). Patient-reported outcome measures (PROMs) showed high heterogeneity, limiting clear conclusions.

**Conclusions:**

CAM demonstrates significant benefits in pain reduction and functional recovery compared to CPM following TKA. However, variability in outcome measures and intervention protocols limits comparability across studies and therefore generalizability. CAM’s requirement for active patient engagement may present practical challenges, while CPM’s standardization allows for easier integration into routine postoperative care.

## Introduction

Early rehabilitation is important after total knee arthroplasty (TKA). Continuous passive motion (CPM) has been employed as a postoperative procedure since the early 1980s, most commonly in the form of passive motion splints applied to the operated leg in conjunction with standard physiotherapy. Despite the fact that CPM has since been employed as a standard treatment in the early rehabilitation phase [1], there is still a lack of evidence to support this assertion. Initially, CPM was assumed to offer advantages for tissue healing and thereby enable earlier mobilization [2].

The procedure has often been evaluated with respect to the ROM as a primary marker of joint function [3–5], which was found to be not significantly advantageous compared to standard physiotherapy. Furthermore, studies analyzed knee swelling and wound healing during CPM usage [6], yielding no positive outcomes and, in some cases, even worse results potentially associated with CPM usage. Overall, the majority of studies, systematic reviews and meta-analyses have failed to demonstrate a clear positive effect or superiority of CPM compared to patients who only received standard physiotherapy [2, 5, 7, 8]. On the contrary, some researchers concluded that study results so far do not provide sufficient evidence to support the routine application of CPM [7], while others discontinued its use entirely in their institutions [6, 9]. Contributing factors may include the limited functionality of such motion devices [10] or the lack of active patient participation in the recovery process [11].

Active motion-based approaches, which rely on the patient’s active involvement and the use of their leg muscles, appear more promising in this context as for instance muscle function, patients’ involvement, and blood circulation are likely to be more facilitated.

The Enhanced Recovery After Surgery (ERAS) Society recommends the earliest possible mobilization for patients following TKA, and studies have demonstrated positive effects of early physiotherapy on functional outcomes, ROM, and length of stay (LOS) [12]. However, the type and duration of physiotherapy interventions vary significantly across the literature.

A preliminary search of Systematic Reviews was conducted and no current or underway systematic reviews on the topic have been identified. The number of studies investigating alternative exercise methods to CPM following TKA remains limited, and even fewer studies directly compare active exercise applications to the CPM method. Among the published studies, the implementation of these exercise approaches varied just as significantly as the selection of endpoints. In contrast, the literature base regarding other knee surgeries is already more comprehensive. For instance, Friemert et al. found that a CAM device, following the principles of closed kinetic chain (CKC) exercises, reduced the proprioceptive deficit reported after an ACL surgery [13]. Drawing from these findings, among others, Fitz et al. hypothesized that potential advantages of CAM include reduced anterior tibial translation, increased tibiofemoral forces, enhanced hamstring co-contraction, and the ability to simulate functional movement patterns [14]. CAM allows patients to actively participate in their rehabilitation process, enabling a more functional recovery that includes neuromuscular activation and promotes an earlier return to daily activities [11].

A similar conclusion was also drawn in a different systematic review, which highlighted the “need for further high-quality studies on supervised exercise therapy programs to achieve better functional outcomes following TKA, particularly in the early postoperative phase” [15].

Therefore, the aim of this meta-analysis was to evaluate whether the addition of CAM provides superior outcomes compared to CPM in early rehabilitation following TKA. Primary outcomes included pain and functional recovery, while secondary outcomes encompassed ROM and PROMs.

## Methods

For this systematic review, the guidelines of the Preferred Reporting Items for Systematic Reviews and Meta-Analyses (PRISMA) and Author Guidelines for Systematic Reviews and meta-analysis in the field of interest were followed [16, 17]. It has been registered in PROSPERO on November 10th, 2024, with the registration number: CRD42024608079.

### Search Strategy

A systematic literature search was conducted in Medline via PubMed, Cochrane Library (including integrated searches across Embase, Cinahl, ICTRP, and Clinical trials.gov) and Epistemonikos for relevant studies by the primary investigator (CS).

The search terms used were 'continuous passive motion,' 'continuous active motion,' 'CPM,' 'slider board,' and 'active heel slide,' combined with the Boolean operator 'OR' among each other and subsequently connected with 'AND' to 'total knee*' to include possible synonyms for TKA. All results from 1984 to February 2025 were considered. ‘CAM’ was only used in the search string with the combination of’continuous active motion’ in the full text, as CAM is used as an abbreviation in diverse contexts.’Cycling’ has been excluded as it requires a flexion of at least 90° in the knee joint.

### Eligibility Criteria

Only randomized controlled trials (RCTs) comparing forms of CAM with CPM in patients following primary TKA were considered for inclusion. Furthermore, they needed to focus on short-term effects in relation to various outcomes. Only peer-reviewed and published studies were included. Studies not available in English or German language or those that could not be effectively translated were excluded. Included studies were required to implement the specified interventions in addition to standard physiotherapy and to utilize at least two measurement time points. The internal validity of studies was examined for thematic relevance and whether the methodology, measurement periods, and execution were appropriately applied; specifically, whether the CAM used involved active flexion and extension of the knee joint (with or without assistive devices). Articles were excluded if they did not compare CAM to CPM treatment, but CAM or CPM with other interventions such as TENS (transcutaneous electrical nerve stimulation), cryotherapy, or other methods. The inclusion criteria were outlined using the PICOS statement, as presented in Table 1.

**Table 1:** PICOS statement

Population	Patients undergoing TKA, at least 18 years old
Intervention	Use of a type of CAM in addition to standard physiotherapy
Comparison	Use of a CPM – device in addition to standard physiotherapy
Outcome	Pain, function, ROM, PROMs
Study types	Randomized controlled trials
Language	English, German

### Study Selection

Based on the predefined inclusion criteria, title and abstract screening was conducted to identify potentially relevant studies. This was followed by a comprehensive full-text review to determine final eligibility. Two reviewers independently screened studies [CS and JL] and duplicates were removed. In terms of disagreement, consensus was achieved through discussion or by a third reviewer [RP].

### Data Extraction

Articles identified were managed in an Endnote v. 20 (Clarivate Analytics, Philadelphia, PA, USA) database and have been exported to JBI Sumari (Joanna Briggs Institute).

Data were extracted from studies by two independent reviewers using the standardized JBI data extraction tool. The extracted data encompassed detailed information on the study populations, trial designs, interventions, application periods, measurement time points, and outcomes relevant to the review question.

### Assessment of Methodological Quality

Eligible studies have been critically appraised by two independent reviewers (CS, JL). For methodological quality in the review, standardized critical appraisal instruments from JBI Sumari were used. Subsequently, the studies included in this review were further assessed for their suitability for meta-analysis, based on the comparability of reported outcomes.

In case of missing or additional data, authors have been contacted for clarification. If the full text was not available or authors did not answer the request, papers have not been included. Any disagreements that arose have been resolved through discussion or with a third reviewer.

### Statistical Analysis

Potential heterogeneity resulting from the inclusion of studies with varying methodological quality was examined based on the risk of bias assessment in JBI SUMARI, including allocation concealment, blinding of outcome assessment and staff, among others. Also, I^2^ was assessed in meta-analysis for statistical heterogeneity. If an outcome of interest was reported in at least two studies, quantitative data analysis for the meta-analysis was conducted. Effect sizes for eligible outcomes were determined using standardized mean differences (SMD), each accompanied by 95% confidence intervals (CIs).

## Results

### Study Characteristics

**Fig. 1:**
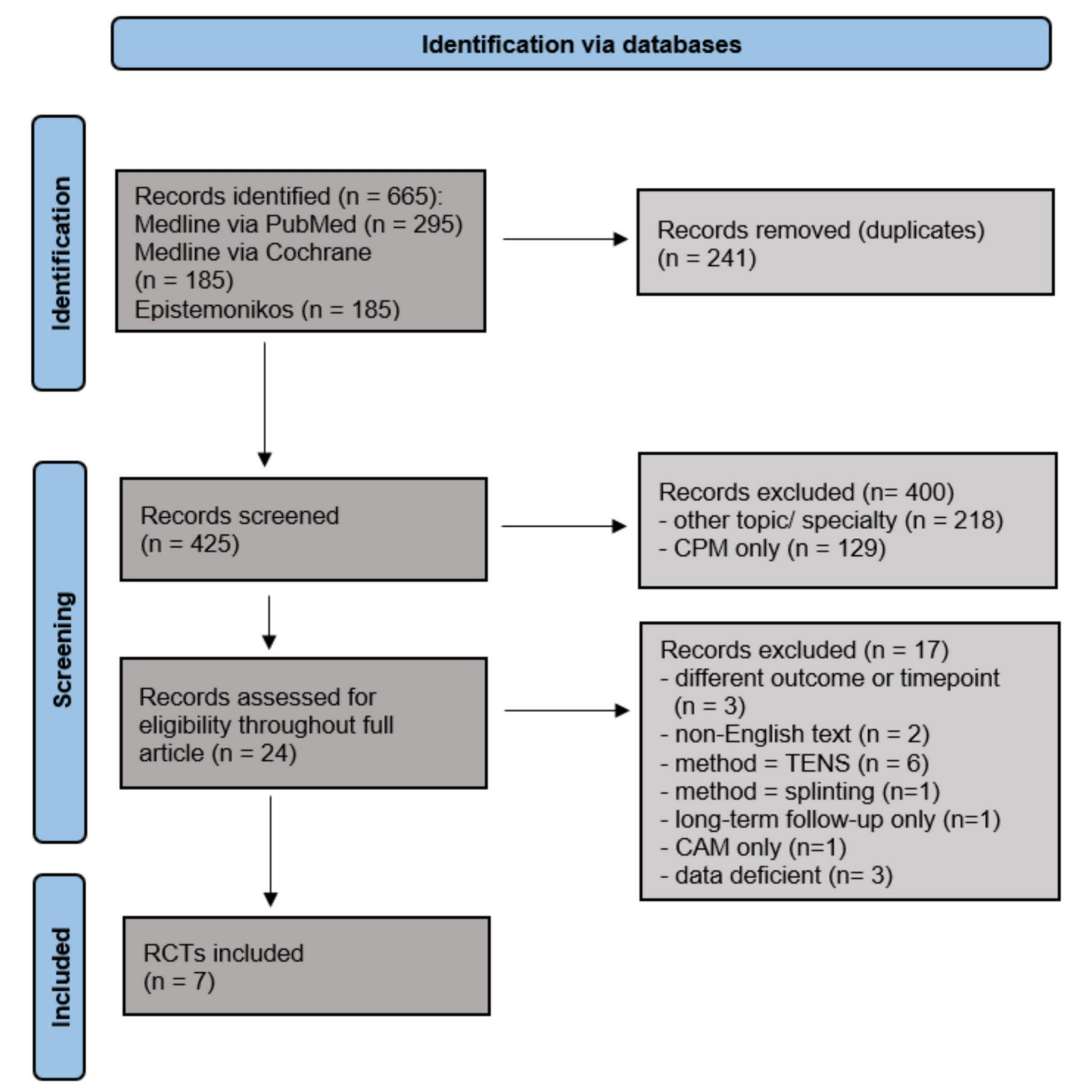
Prisma 2020 flow diagram for systematic review

A total of 425 titles and abstracts were initially screened. After the initial screening phase, 400 records were excluded for not meeting the inclusion criteria, leaving 24 RCTs for full-text assessment. Of these 24 RCTs, seven met the eligibility criteria for inclusion. The results are presented in the PRISMA Flow Chart in Fig. 1.

**Fig. 2:**
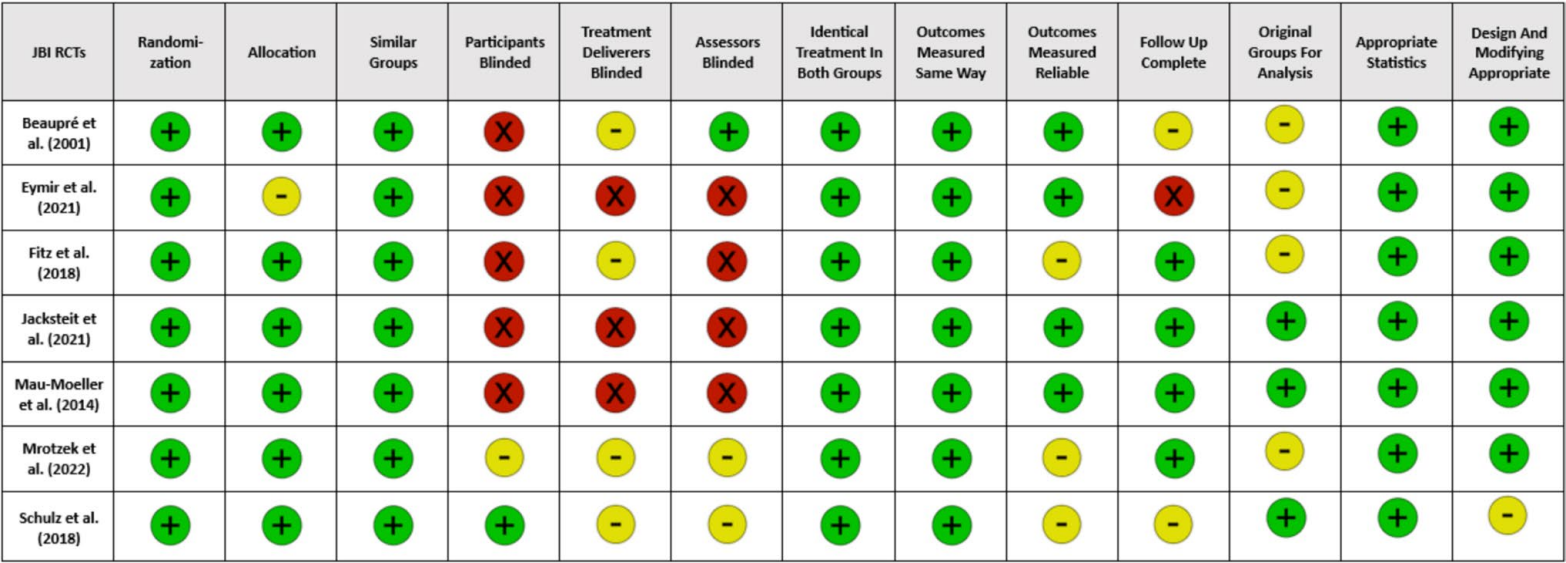
Results of JBI critical appraisal

Among the final selection of RCTs, one was a pilot study [18]. This results in a total inclusion of 501 patients, ranging in study size from 31 to 120 patients. In six studies, patients scheduled for a primary, unilateral TKA were included, while one study included only medial but both UKA and TKR [14]. Study characteristics are shown in Table 2. Baseline patient characteristics are shown in Table 3. The quality assessment of the studies was evaluated with the JBI critical appraisal tool, being presented in Fig. 2.

**Table 2:** Study characteristics

Publication	Outcomes	Timing and duration (CPM)	Timing and duration (CAM)	Outcomes assessment
Location		Intervention procedure Control Group (CPM)	Intervention procedure (CAM)	
Beaupré et al. 2001	aROM, pain, KOOS, adverse events	3 × 2 h/d + SE	2 × 10 min/d + SE	1. preop. 2. 5–7 days postop. 3. 3 months postop. 4. 6 months postop
Canada		"CPM machine"	slider board exercise	
Eymir et al 2021	HSS, knee proprioception, pain, active ROM, knee circumference, LOS, time for achieving straight leg raise actively, time for achieving 70° knee flexion	2 × 30 m/d—start speed: 2°/s -10°/s, angle increase: 5–15°/d	2x/ d a 3 sets/20 reps (20 × Flex-hold 5 s- Ext/ 1 min break after each set)	1. 1–2 d preop. 2. at discharge 3. 3 months postop
Turkey		"CPM machine"	Active heel-slide	
Fitz et al 2018	functional outcome measures (kinesthesia, quadriceps strength, coordination), SF-36, KSS, WOMAC, narcotic consumption	4 h/d for 3 weeks	3 × 20 m/d for 3 weeks	1. 1–2 weeks preop. 2. 4 weeks postop
MA, USA		bicycle with linear motion	CAMoped	
Jacksteit et al. 2021	active knee flexion range of motion (ROMFlex). 2nd: active knee extension ROM, swelling, pain, CRP, Qol, physical activity, TUG, stair-climbing performance, quadriceps muscle strength	3 × 30 min/ d	CAMuni: 3 × 30 min/ d CAMbi = 3 × 30 min/day op. side + 1 × 30 min kontralat. Side (warm-up = 20 rep. @ restistance level 0–2; 5 sets @ level 3; 20 reps @ level 0)	1. 1 d preop. 2. 9 d postop 3. 3 months postop
Germany		Kinetec® Optima TM S3	CAMoped	
Mau-Moeller et al. 2014	1st: passive ROMflex, 2nd: active ROMflex, active & passive ROMext, static postural control, physical activity, pain, LOS, clinical, SF-36, HSS and WOMAC scores	2 × 30 min/ d (0–120°)	2 × 30 min /d (0–90°)	1. preop. 2. 1 d before discharge 3. 3 months postop
Germany		Kinetec® OptimaTM S3 and S4	sling exercise	
Mrotzek et al. 2022	OKS, pain, passive ROM	2x/d for 30–45 min	2x/d à 5x (20 reps + 5 min break) = total of 30–45 min	1. preop. 2. 7 d postop. 3. 8 d postop. 4. 8 weeks postop
Germany		"CPM motor rail"	single-joint hybrid assistive limb (HAL-SJ)	
Schulz et al 2018	Pain, KOOS, active ROM, adverse events	ARTROMOT Active-K	ARTROMOT Active-K	1. preop. 2. 4–5 d postop. 3. 8–9 d postop. 4. 30 days after outpatient care
Germany		n.a	n.a

**Table 3:** Patient characteristics

Author/ Year	Interventional laterality	No. of participants		Sex F/M		Mean Age ± SD		Inclusion criteria	Exclusion criteria
		Control	Exp	Control	Exp	Control	Exp		
Beauprè et al. 2001	Unilateral	40	40	21/19	20/20	68 ± 9	68 ± 9	primary bilateral TKA	Revision knee surgery, unicondylar TKA
Eymir et al. 2021	Unilateral	55	58	50/5	49/9	68,9 ± 8,3	68,9 ± 8,9	> 30 years old, unilateral primary TKA, BMI < 40 kg/m^2^	Previous orthopedic, neurological, cardiac disorder or surgery that causes gait disturbance
Fitz et al. 2018	Unilateral	45	38	31/14	20/18	64,7 ± 9,2	61,5 ± 8,8	> 18 years old, unilateral primary medial TKA or UKA	Bilateral UKA/TKA, arthritis on contralateral side
Jacksteit et al. 2021	Unilateral	22	22	14/8	13/9	68,45 ± 9,07	67,36 ± 9,61	50–80 years old, BMI < 40 kg/m^2^; primary TKA	Mini-Mental State Examination score < 25, musculoskeletal and neurological disorders that limit physical function, metabolic bone disease, a surgery planned within the next 12 months, pain or functional restrictions
	Bilateral	22	22	14/8	14/8	68,45 ± 9,07	68,55 ± 8,94	s.a	s.a
Mau-Moeller et al. 2014	Unilateral	19	19	9/10	7 /12	67,1 ± 8,8	68,8 ± 8,0	50–80 years old BMI < 40 kg/m^2^ primary TKA	Musculoskeletal and neurological disorders that limit physical function
Mrotzek et al. 2022	Unilateral	16	15	8/8	6/9	59,31 ± 6,89	58,13 ± 5,72	< 79 years old BMI < 40 kg/m^2^ Kellgren-Lawrence-score > / = 2, TKA due to osteoarthritis or posttraumatic	Paralysis of the lower extremity, Parkinson's disease, muscular dystrophies, skin diseases aggravated by the application of electrodes, massive edema of the lower extremity with a maximum thigh circumference > 70 cm, pain that makes a conventional rehabilitation impossible, non-device related revision surgery, pacemaker
Schulz et al. 2018	Unilateral	25	25	14/11	12/13	71 ± 8	69 ± 8	18 > x < 80 years old Gonarthrosis grade VI, TKA	For whom motion therapy was contraindicated (e.g., in case of acute joint inflammation or spasticity)

### Outcome Analysis

#### Pain

**Fig. 3:**
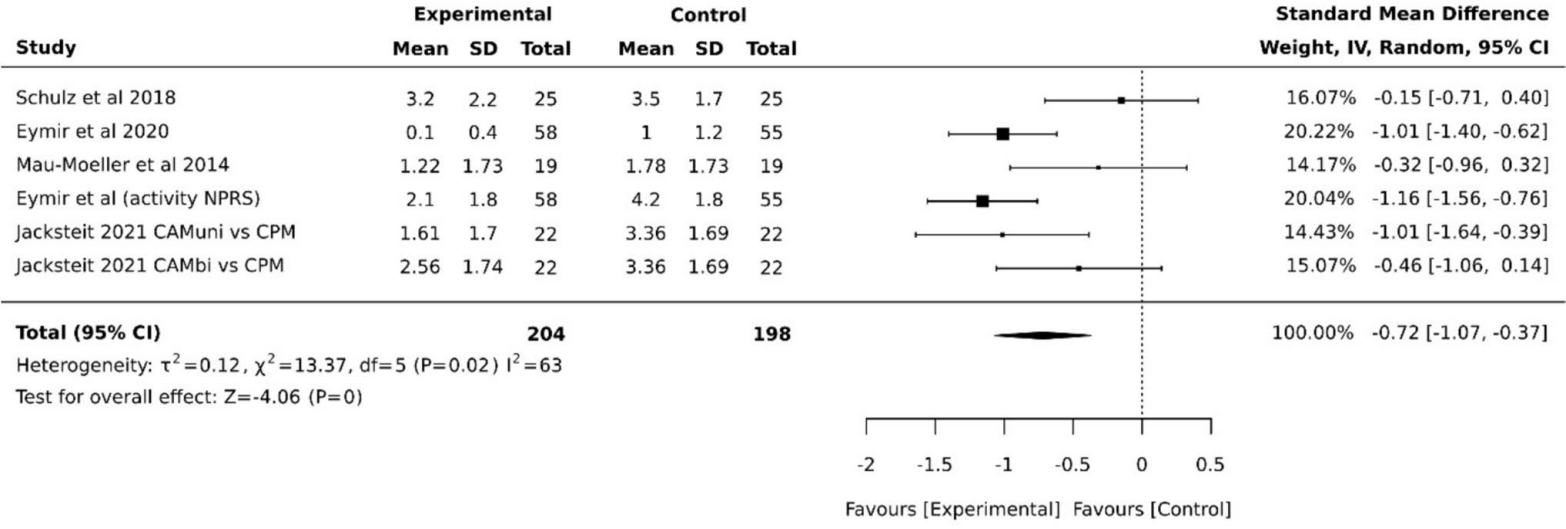
Forest plot diagram of pain at posttest (postoperative, around discharge)

**Fig. 4:**
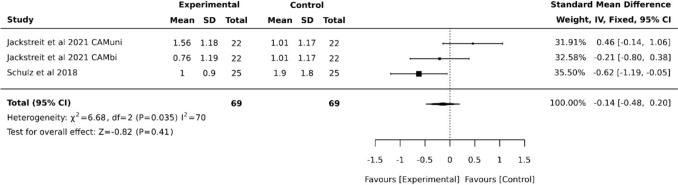
Forest plot diagram of pain at follow-up (3 months postoperative)

Five studies assessed pain using the Visual Analog Scale (VAS) or Numeric Pain Rating Scale (NPRS) at least at one follow-up time point [10, 11, 19, 20]. In four of these studies, the results were reported as specific values, whereas one study presented only difference values, which was therefore excluded from the meta-analysis [18]. The results of the meta-analysis are shown in Fig. 3 and indicate high heterogeneity at the time of discharge (5–9 days postoperatively, post-test) and a benefit for the CAM groups, with an SMD of −0.72 (95% CI −1.07, −0.37; *p* = 0). Included in this analysis were both subgroups (CAMuni, CAMbi) from Jackstreit et al. compared to CPM, as well as the dataset from Eymir et al. on "pain in motion." Therefore, strictly speaking, only four studies were ultimately integrated. Beauprè et al. assessed pain only as part of the Western Ontario and McMaster Osteoarthritis Score (WOMAC score), which was excluded from the analysis due to the lack of reported significant differences [21]. At follow-up time point, the two eligible studies (including an additional subgroup) [19, 20] showed high heterogeneity and no significant advantages for CAM, with an SMD of −0.14 (95% CI −0.48, −0.20) (Fig. 4). CAM refers to the experimental group and CPM to the control group throughout all figures.

#### Function

Most of the studies represented function primarily throughout various PROMs. Four studies utilized the Short-Form- 36 Health Survey (SF-36), three used the WOMAC score, two employed the Hospital for Special Surgery (HSS) knee score, and one applied the Knee Society Score (KSS). Due to the heterogeneity of the PROMs used, these were not included in the synthesis. Additionally, the Sit-to-Stand Test [14], stair-climbing performance, isometric maximal voluntary torque (iMVT) [19], and four different activities within the Iowa Level of Assistance Scale (ILAS) [11] were used as measurable parameters, but only in individual studies. Significant differences in favor of the CAM group at the first postoperative measurement time point were observed in the Sit-to-Stand Test (*p* = 0.015), stair climbing (*p* = 0.038) within the ILAS [11], and stair-climbing performance (*p* = 0.016) [19]. At follow-up, only Fitz et al. reported a benefit for CAM in the Sit-to-Stand Test (*p* = 0.017).

**Fig. 5:**
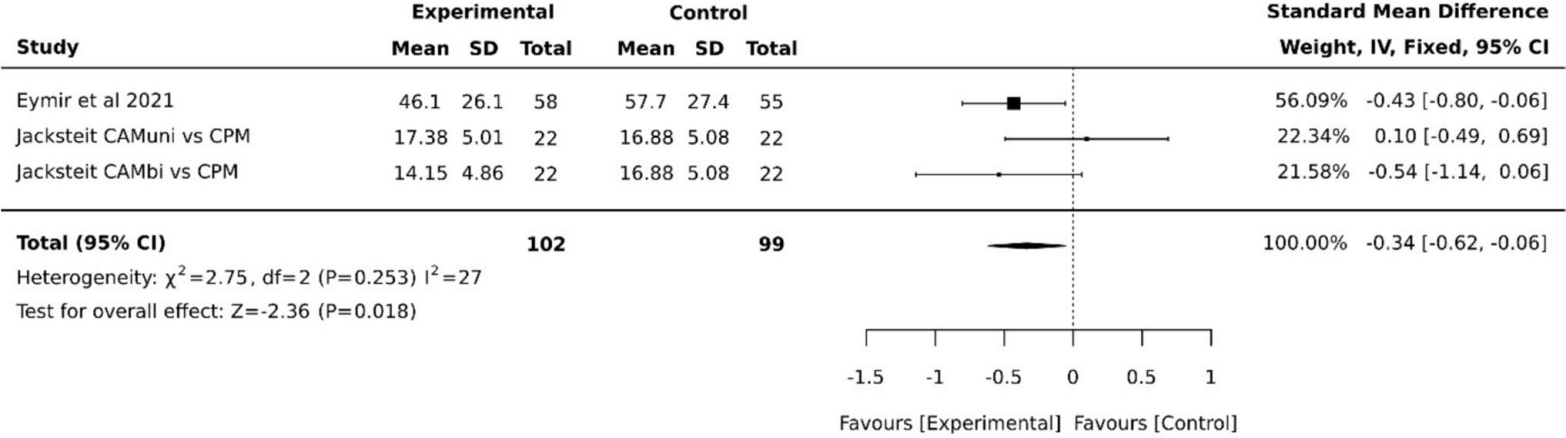
Forest plot diagram of function via timed-up-and-go Test (TUG) at posttest (postoperative, around discharge)

The Timed-Up-and-Go Test (TUG) was used as a parameter for function in the meta-analysis based on data from two studies [11, 19]. Despite relatively high heterogeneity, the results indicated a benefit for CAM, with an SMD of −0.34 (95% CI −0.62, −0.06) (Fig. 5).

#### Active Range of Motion

**Fig. 6:**
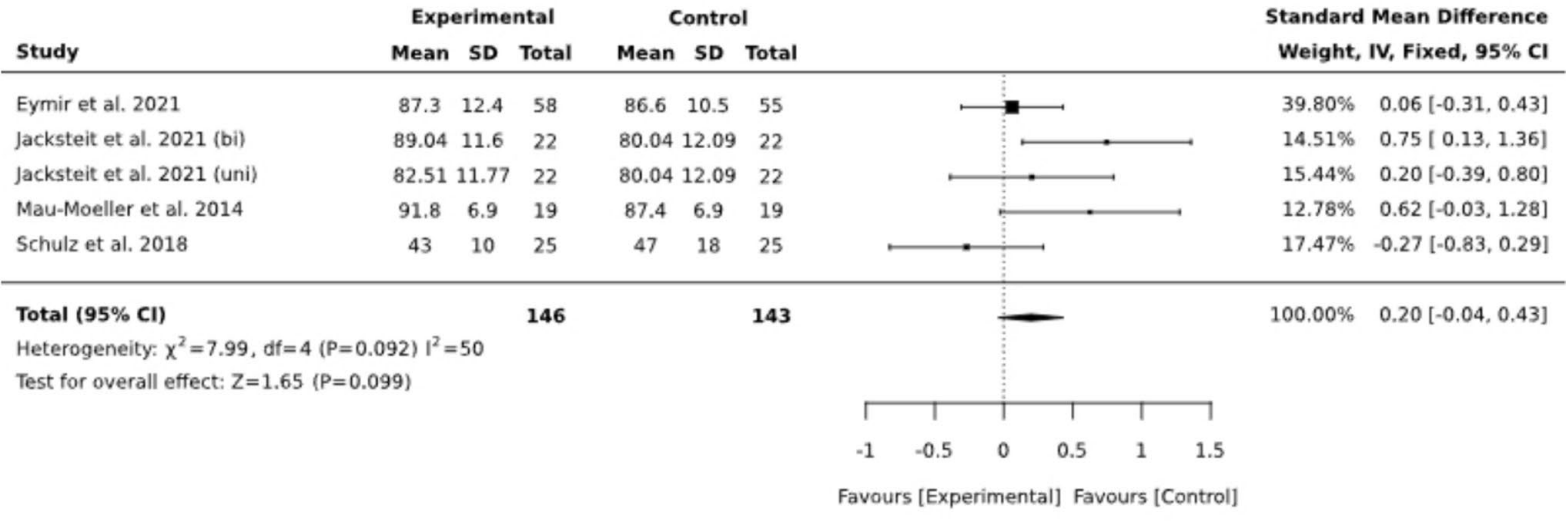
Forest plot of aROM at posttest (postoperative, around discharge)

**Fig. 7:**
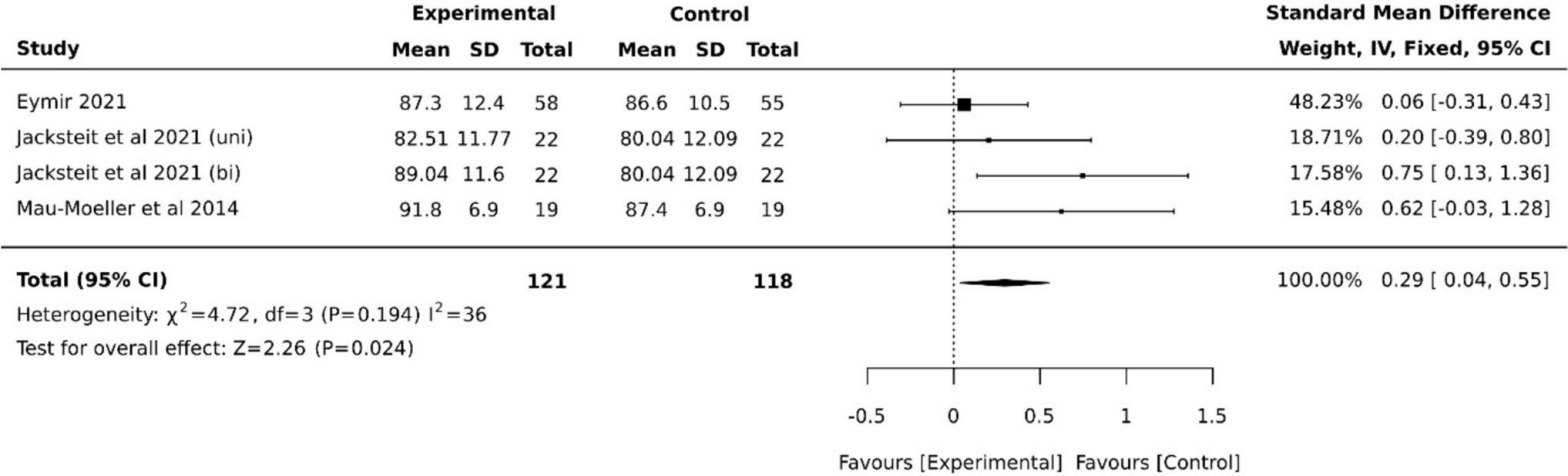
Forest plot diagram of subgroup analysis of aROM at posttest (postoperative, around discharge)

Short-term active ROM (aROM) was reported in four studies with 133 patients, including both uni- and bilateral CAM groups from Jacksteit et al. No difference was observed between the randomized groups, SMD = 0.20 (95% CI −0.04, 0.43). Statistically significant heterogeneity existed among the studies (I^2^ = 50) (Fig. 6). Since one study deviated significantly in this analysis, a subgroup analysis was conducted, excluding this group for comparison, resulting in an outcome favoring CPM, SMD = 0.29 (95% CI 0.04, 0.55) and lower heterogeneity (I^2^ = 36) (Fig. 7).

**Fig. 8:**
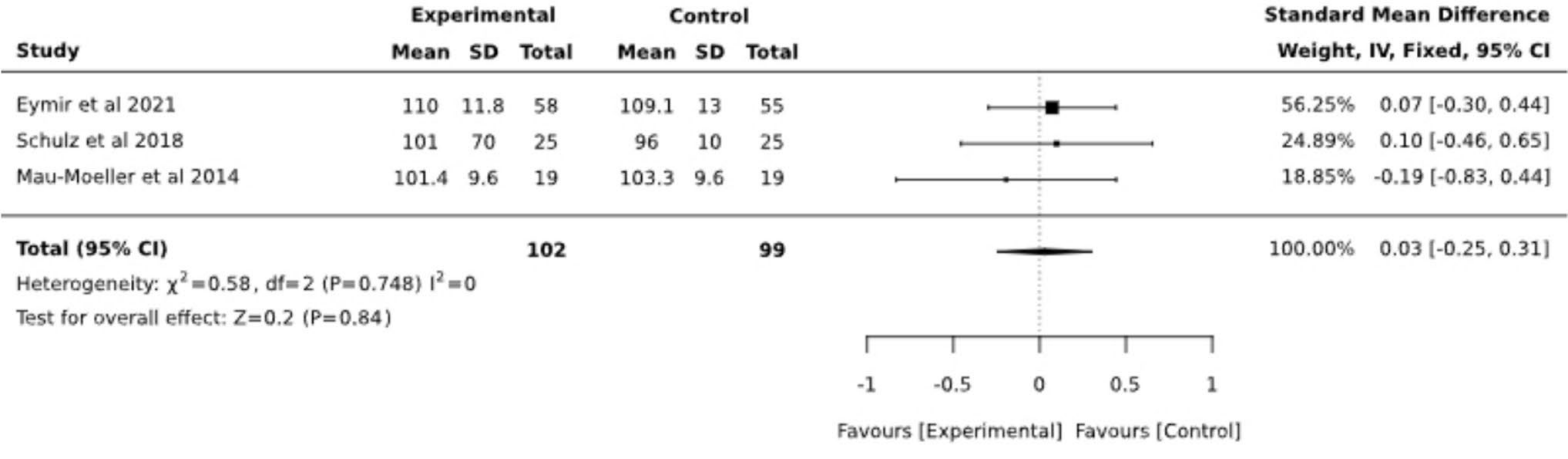
Forest plot diagram of aROM at follow-up (3 months postoperative)

For illustration purposes, analysis was conducted for follow-up, including three studies yielding an SMD = 0.03 (95% CI −0.25, 0.31) with no significant heterogeneity (I^2^ = 0) (Fig. 8).

#### Passive Range of Motion

**Fig. 9:**
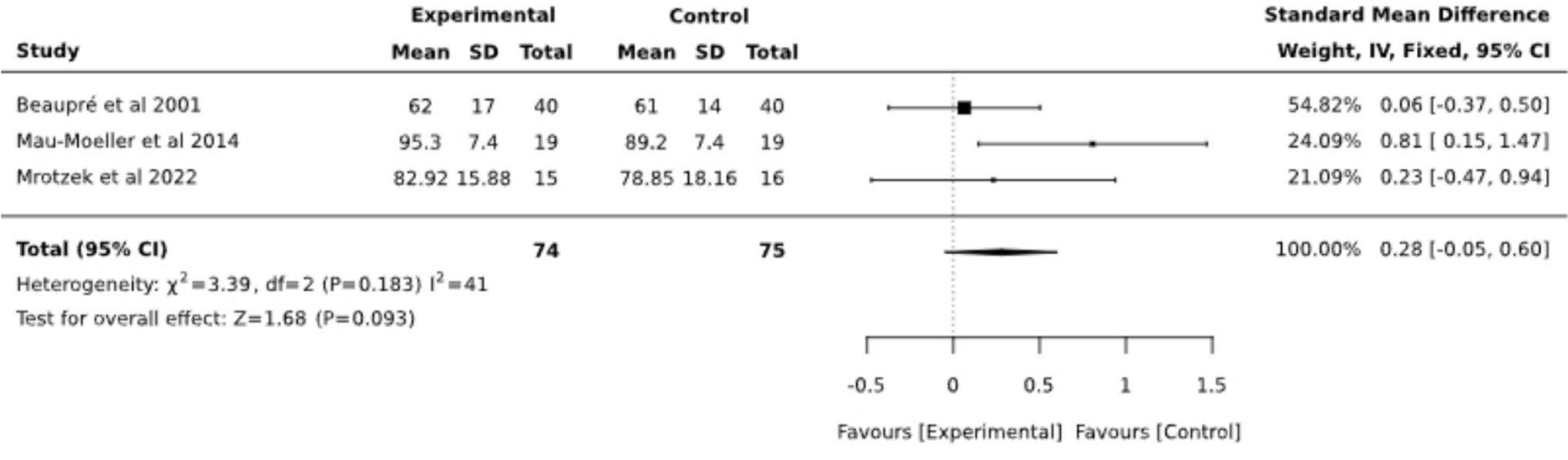
Forest plot diagram for passive range of motion (pROM) at posttest (postoperative, around discharge)

**Fig. 10:**
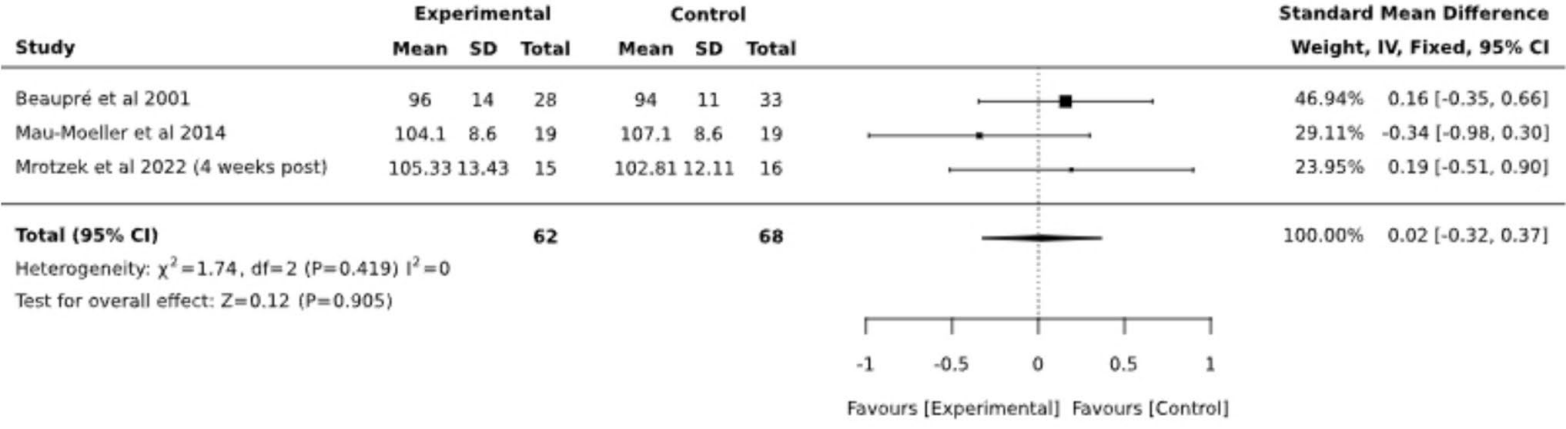
Forest plot diagram for pROM at follow-up (3 months postoperative)

Postoperative passive ROM (pROM) involving 75 patients was analyzed in a total of 3 trials [10, 18, 21]. The overall results (SMD 0,28; CI95%: −0.05, 0.60) suggested no significant effect (Fig. 9). For comparison purposes, analysis was performed for the follow-up timepoint, resulting in no significant difference between the same groups, SMD = 0.02 (95% CI −0.32, 0.37) (Fig. 10).

#### Patient Reported Outcome Measures

Details about PROMS were available in all seven studies, each utilizing different PROM tools. This includes the KSS [14], KOOS [20], and Oxford Knee Score [18], each used in one study, as well as the HSS Knee Score, which was used in two studies [10, 11]. Furthermore, the WOMAC was utilized in three studies [10, 14, 21], and the SF-36 in four of the seven studies analyzed [10, 14, 19, 21]. Due to the heterogeneity of the PROMs used, these were not included in the synthesis.

### Descriptive Summary of Included RCT’s

#### Interventions

Active and passive interventions slightly differ in most of the studies. A CAM variant with assistive devices was utilized in six studies: two used the CAMOped brace [14, 19], one the ARTROMOT Active-K (Ormed GmbH) [20], one employed "sling exercise" in the form of a "tubular bandage" [10], another used a "slider board" [21], and one implemented the "single-joint assistive limb" (HAL-SJ) [18]. Only the study by Eymir et al. applied CAM without additional equipment, relying solely on active movement in the form of an "active heel-slide" performed on the bed surface [11].

For CPM devices, two studies used the Kinetec Optima TM S3 or S4 (AbilityOne Kinetec S.A., Tournes, France) [10, 19], four studies did not specify the device beyond describing it as a "CPM machine" or a "bicycle with linear motion" [14], while Schulz et al. also utilized the ARTROMOT Active-K [20]. Thus, this study represents the only one employing a single device (brace) for both intervention modalities.

#### Outcomes

The studies demonstrated high variability in the primary and secondary outcome parameters. Regarding the timing and frequency of measurements, all studies conducted a preoperative measurement to establish baseline values. The number of postoperative measurements ranged from at least one to a maximum of three assessments. In six out of seven studies, the first postoperative measurement was reported to occur around the day of discharge (approximately 4–9 days postoperatively). The follow-up duration varied from 4 weeks to 6 months.

## Discussion

The primary finding of this review was that CAM can result in significant pain reduction as well as in better functional outcomes in patients following TKA when compared to CPM. Some studies already had significant results in their setting; some showed no significant effect but a trend towards CAM. According to the Outcome Measures in Rheumatology Clinical Trials (OMERACT) Total Joint Replacement (TJR) core domains [22], these two parameters are among the most important core domains (and should be included in every RCT) [23]. Therefore, they were considered clinically relevant outcomes.

The results of the meta- and subgroup analysis for postoperative self-reported pain indicate a benefit for the CAM groups. Both subgroups (unilateral and bilateral use) from Jacksteit et al. were included, further reinforcing the positive trend in favor of CAM. It is important to note the variation in the timing of post-tests among the included studies, which was influenced by highly variable discharge times ranging from four days [20] to nine days [19]. This variability, for example, resulted in a shorter intervention duration and healing phase in the study by Schulz et al., which might have influenced the favorable active ROM outcomes reported for the CAM group. Nonetheless, the fact that such improvements were observed despite the reduced exposure highlights the substantial effect of CAM during the early postoperative phase and reinforces the overall evidence in favor of the active intervention. Although both outcome groups from Eymir et al. were included, as pain was measured both at rest and during movement, the results are both clear and statistically significant [11]. A possible explanation for these findings could be the increased blood flow during the exercises due to muscle contractions, which may promote a more efficient oxygen supply to the involved muscles and joints, thereby contributing to pain relief [24]. Additionally, movement activates the endogenous opioid system through the release of β-endorphins, which exert peripheral and central analgesic effects via µ-opioid receptors, with the hypothalamus playing a key role by activating descending nociceptive inhibitory mechanisms [24]. Furthermore, CAM exercises align with the principles of closed kinetic chain (CKC) exercises, which enhance the activity of both agonist and antagonist muscles compared to open kinetic chain (OKC) exercises, such as those employed with CPM [14].

Regarding the second domain, “physical function,” the wide range of representation options within the included studies highlights the extent to which the term is still variably defined and, consequently, assessed. Physical function in this context is predominantly assessed using PROMs rather than performance-based outcome measures (PBOMs) [23]. Only two studies utilized the TUG test, which is classified as a PBOM and has been described by Adriani as "one of the most well-validated PBOMs" [23]. While the meta-analysis indicated a benefit for CAM, its findings should be interpreted with caution due to the noticeably higher values reported for both the control and intervention groups in Eymir et al. compared to the subgroups in Jacksteit et al. [19]. However, since the measurement criteria in both studies were described as nearly identical (except for a 1 cm height difference in the chair used), no relevant reasons for these discrepancies could be identified. As another PBOM, Fitz et al. used the Sit-to-Stand Test and were able to reinforce the idea that initiating early activation of the operated leg facilitates faster functional recovery, as evidenced by the significantly better results in the CAM group [14]. Overall, only three of the seven included studies utilized PBOMs [11, 14, 19], contrary to the current recommendation to include at least one in the clinical assessment of every patient [23].

Jacksteit et al. demonstrated the highest number of PBOMs and physical activity measurements (sit-to-stand transitions and steps) and were the only study to investigate a subgroup that additionally used a CAM brace unilaterally for the contralateral, i.e., non-operated side [19]. They were able to confirm their hypothesis that active movement, particularly of both legs, offers an advantage in terms of functional endpoints and physical activity. These results may be attributable to the so-called "Cross-Education" (CE) effect, which has been shown to lead to contralateral strength gains following unilateral strength training [25, 26]. This phenomenon has since been extensively studied and has proven to be highly consistent, particularly with regard to dynamic and eccentric contractions [27]. The effect is primarily muscle-specific and mainly affects contralateral homologous muscles, but it can also involve synergists to a lesser extent. According to the authors, the CE effect "could be considered as an adjunctive treatment, particularly for unilateral orthopedic conditions" [27]. This CE effect could potentially play a role in explaining the better outcomes observed in the CAM group, as at least an active contraction of the non-operated leg occurred during their interventions, which may have positively influenced the contralateral (operated) side [28].

In contrast to the other six studies, Mrotzek et al. used an active-assistive movement form with the HAL-SJ for their intervention group, making their study open to discussion [18]. Since both, the range of motion and the device itself were autonomously and neurologically controlled by the patient's bioelectrical muscle signals, we included this study in our analysis; furthermore, to broaden the results.

Contrary to the OMERACT recommendations, ROM is still commonly used as a primary parameter for evaluating the success of TKA, often based on the assumption that a minimum degree of knee flexion is essential for activities of daily living or hospital discharge [7]. The exact threshold of required knee flexion range of motion remains debated in the literature [2, 29, 30]. According to Maniar et al., even an increase in knee flexion range of motion of more than ten degrees is required to justify the additional time and costs associated with a motion-based intervention following TKA [6]. Compared to the CPM treatment in the study by Jacksteit et al., the CAMbi intervention led to an improvement in active knee flexion range of motion of 9.0° at the post-test and 6.3° at follow-up, which also indicates a clinically significant difference in terms of ROM [19]. In their study on an app-based training program, Hardt et al. demonstrated that aROM, unlike that achieved through CPM, also depends on muscle function and strength [31]; in line with this, the aROM outcomes in their intervention group were significantly better than those in the control group. Eymir et al. also suggest that functional skills such as rising from a chair or walking depend more on lower limb strength than on the ROM achieved [11]. These findings support the notion that the active contraction of the affected limb’s musculature is more critical than the extent of joint movement itself, thereby reinforcing the call for identifying the most appropriate and standardized outcome measures in the evaluation of TKA rehabilitation [32]. As Reynaud et al. (2020) also evaluated [33], the wide range of outcomes that have been examined in patients after TKA, as well as the different measurement instruments used to assess these outcomes, is the most striking finding of our review.

Intervention durations varied considerably across studies. For example, Fitz et al. applied CPM for four hours daily, while CAM was limited to three 20-min sessions only [14]. Beaupre et al. reported an even greater imbalance—six hours of CPM versus just 20 min of CAM per day [21]. Other studies used more comparable durations, though one failed to report timing altogether [20]. These inconsistencies limit comparability and raise questions about protocol design. However, the significant difference in term of treatment time per day underlines the efficiency of CAM.

Additionally, as standard physiotherapy—routinely implemented in the acute postoperative phase—already includes active exercises [10, 11, 14, 19], the added value of passive treatments is debatable. Notably, in Beaupre et al., the CAM intervention (slider board) was already part of the physiotherapy program, potentially confounding the results [21].

A further aspect worth noting are the results reported by Schulz et al. regarding e.g., aROM, which appear questionable as outcome measurements were conducted as early as postoperative day (POD) 4 or 5. This considerably earlier assessment, compared to other studies with later discharge dates and follow-up time points, may have influenced the outcomes. In fact, the markedly inferior results observed in this study—compared to other included trials—are likely attributable to the premature timing of the evaluation, which may not adequately reflect functional recovery. To account for this discrepancy, a subgroup analysis was conducted, excluding this data to reduce heterogeneity and enhance the comparability of results.

This meta-analysis faces some limitations that must be considered when interpreting its findings. Most notably, there was substantial heterogeneity in outcome measures, timing and frequency of assessments, and the duration and execution of interventions—greatly limiting comparability across studies. This is partly due to the varying implementation of CAM, ranging from the use of braces like CAMOped® to active leg exercises without assistive devices. Consequently, both the mode and duration of interventions differed significantly. CPM was also applied using different or unspecified devices.

Regarding pain assessment, only Eymir et al. distinguished between pain at rest and during movement [11]. No stratification by study quality was performed, as methodological details were partially insufficient or inconsistently reported.

## Conclusion

The meta-analysis supports the idea that CAM after TKA can lead to statistically significant improvements in postoperative pain and activity level. While the evidence supporting functional and proprioceptive benefits is promising, data regarding improvements in range of motion remain inconsistent and overall limited. These findings underline the increased value of active rehabilitation strategies.

## Data Availability

The datasets used and/or analyzed during the current study are available from the corresponding author on reasonable request.

## Abbreviations

aROM: Active range of motion
CAM: Continuous active motion
CE: Cross education (effect)
CKC: Closed kinetic chain
CPM: Continuous passive motion
ERAS: Enhanced recovery after surgery
HAL-SJ: Single-joint assistive limb
ILAS: Iowa Level of Assistance Scale
KOOS: Knee Injury and Osteoarthritis Outcome Score
KSS: Knee Society Score
OKC: Open kinetic chain
OMERACT: Outcome measures in rheumatology
PBOM: Performance-based outcome measure
pROM: Passive range of motion
PROM: Patient-reported outcome measures. (Note: PROM can also mean passive range of motion depending on context)
ROM: Range of motion
SF-36: Short-Form 36 Health Survey
TENS: Transcutaneous electrical nerve stimulation
TJR: Total joint replacement
TKA: Total knee arthroplasty
TUG: Timed up and go
WOMAC: Western Ontario and McMaster Universities Osteoarthritis Index
